# Supplementary figures and images for: Tyrosine phosphorylation tunes chemical and thermal sensitivity of TRPV2 ion channel
Source: eLife. 2022 Jun 10;11:e78301. doi: 10.7554/eLife.78301 (PMC9282855; doi:10.7554/eLife.78301)

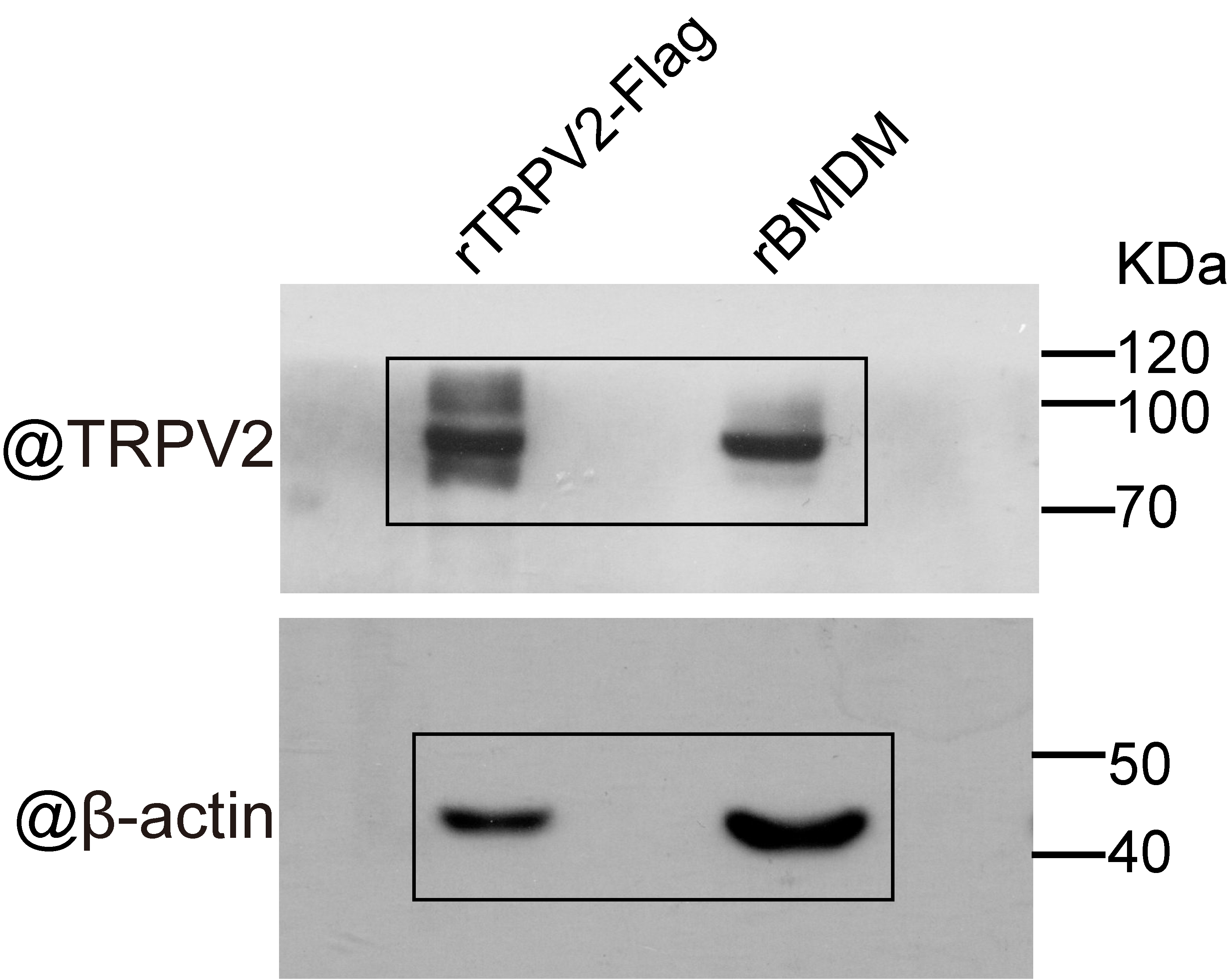

Supplement: Figure 1—figure supplement 1—source data 1. [file elife-78301-fig1-figsupp1-data1.zip › Figure 1 ¿C figure supplement 1-data source 1.tif]

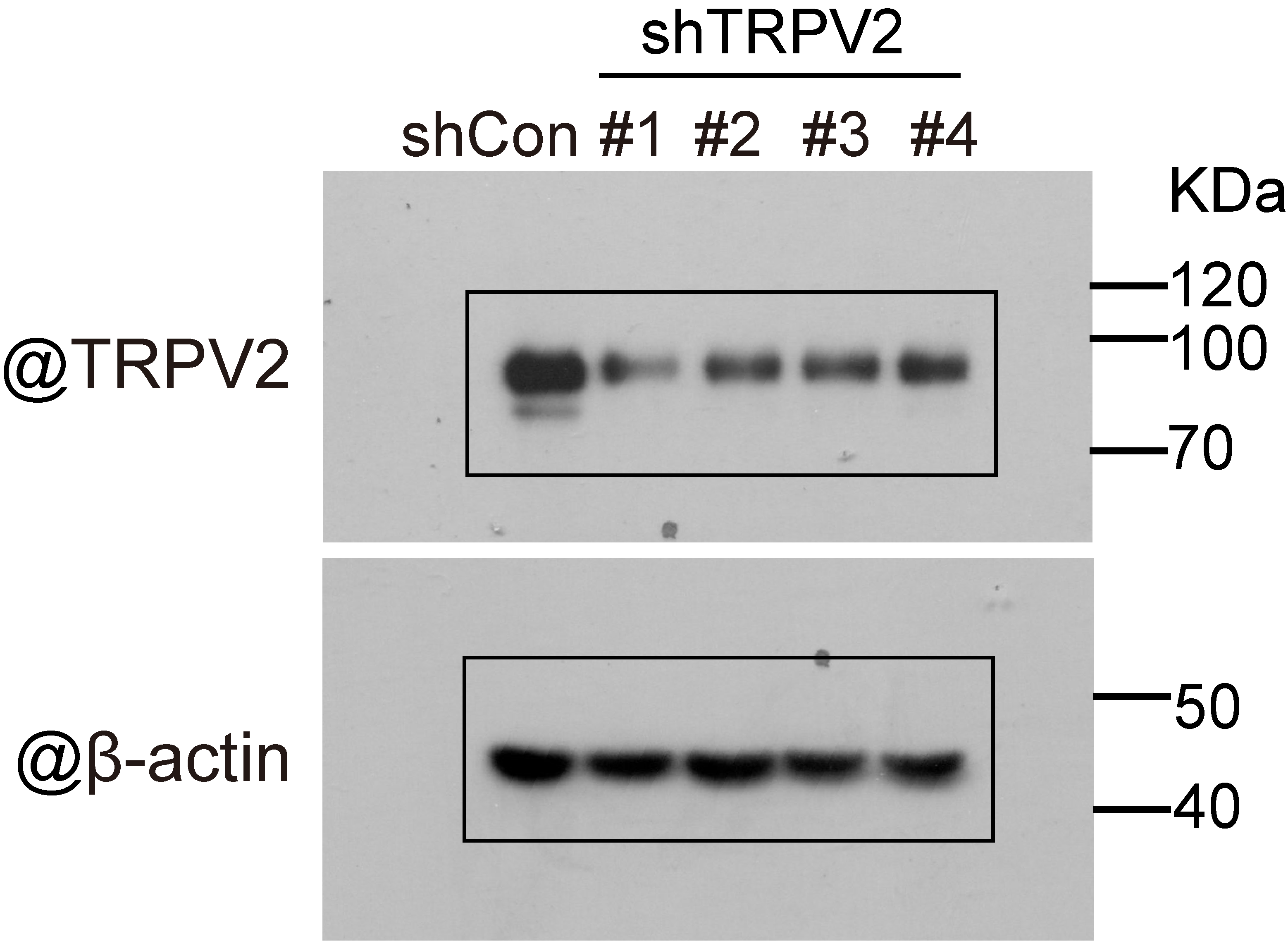

Supplement: Figure 1—figure supplement 1—source data 2. [file elife-78301-fig1-figsupp1-data2.zip › Figure 1 ¿C figure supplement 1-data source 2.tif]

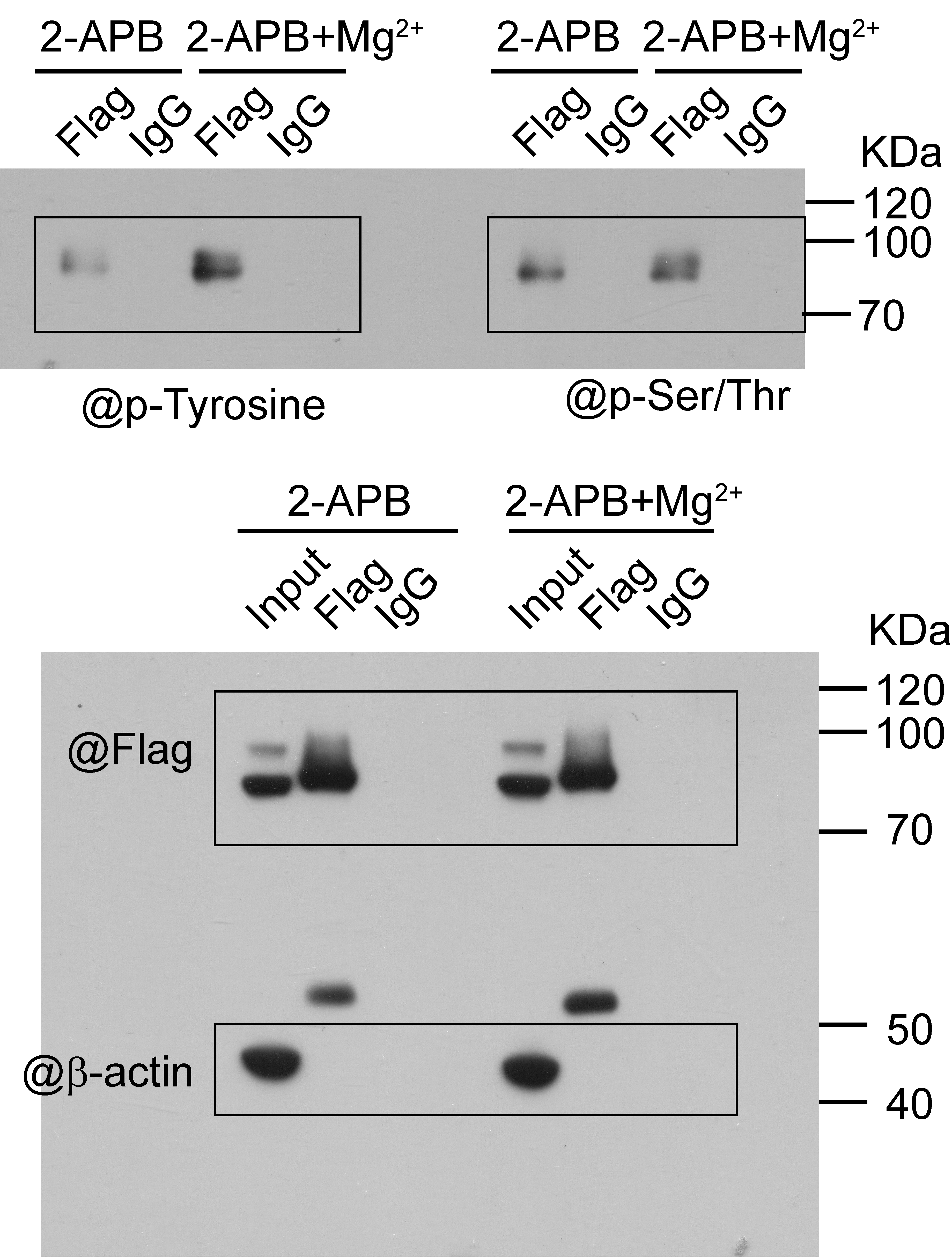

Supplement: Figure 3—source data 1. [file elife-78301-fig3-data1.zip › Figure 3 ¿C data source 1.tif]

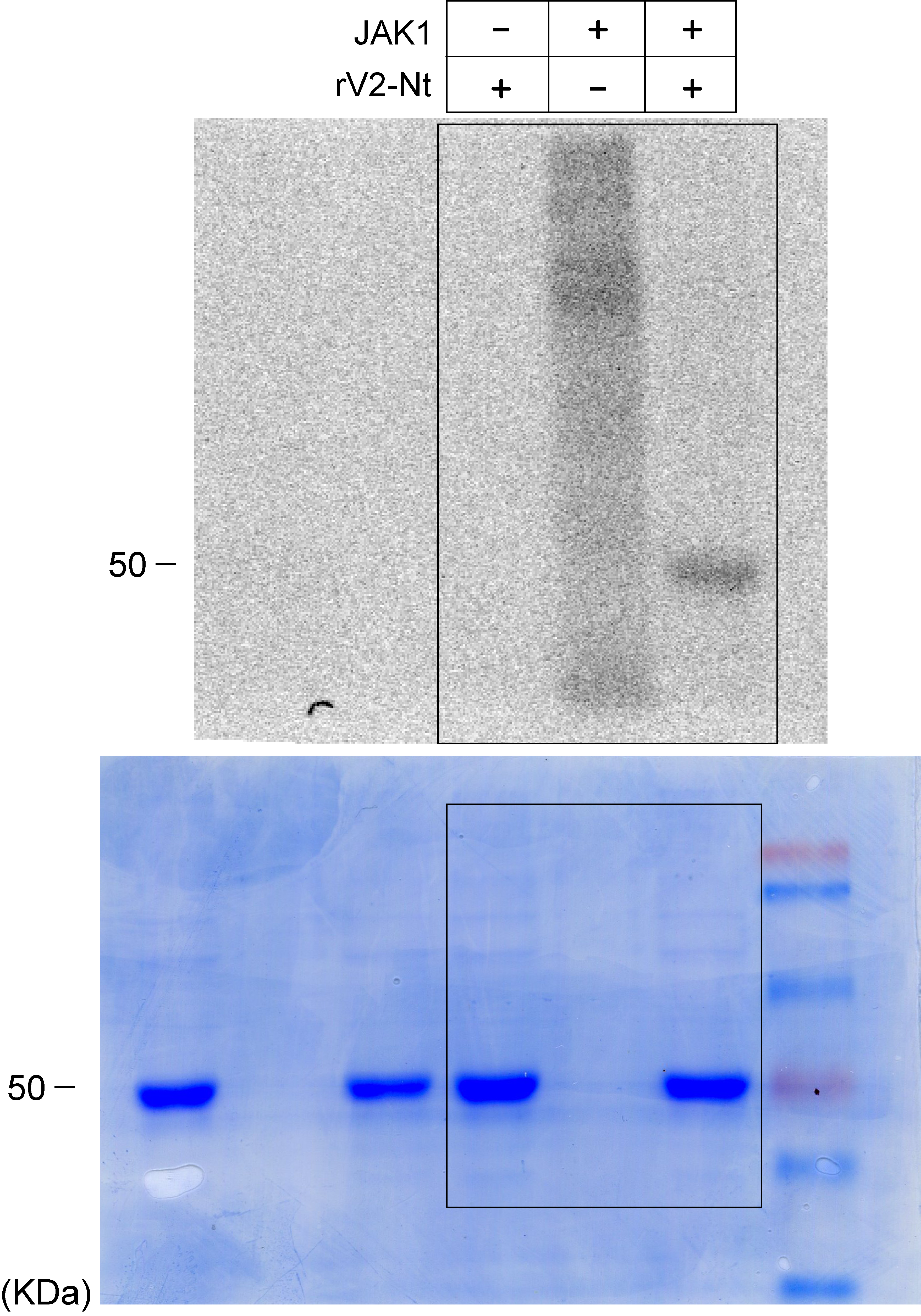

Supplement: Figure 3—source data 2. [file elife-78301-fig3-data2.zip › Figure 3 ¿C data source 2.tif]

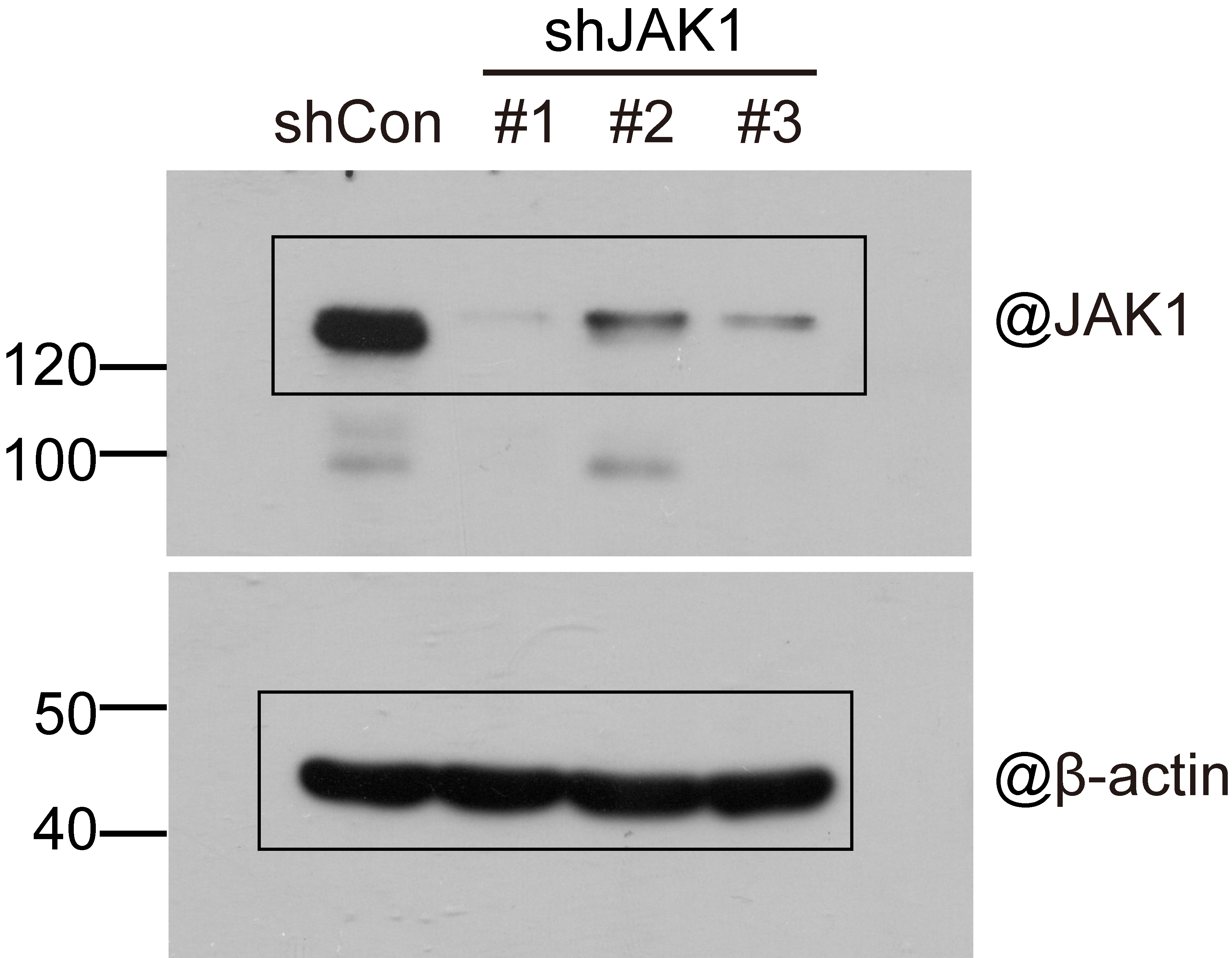

Supplement: Figure 3—source data 3. [file elife-78301-fig3-data3.zip › Figure 3 ¿C data source 3.tif]

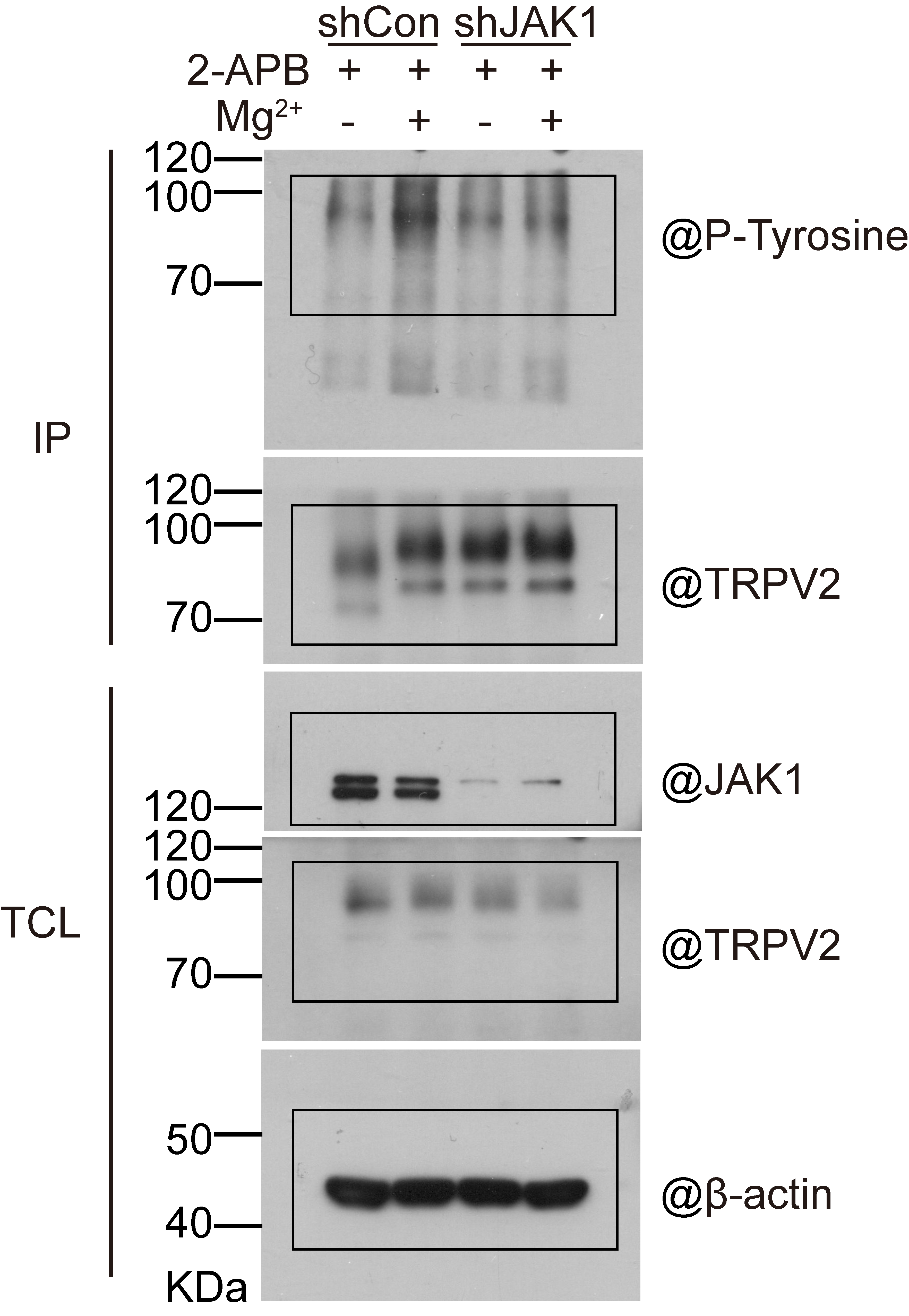

Supplement: Figure 3—source data 4. [file elife-78301-fig3-data4.zip › Figure 3 ¿C data source 4.tif]

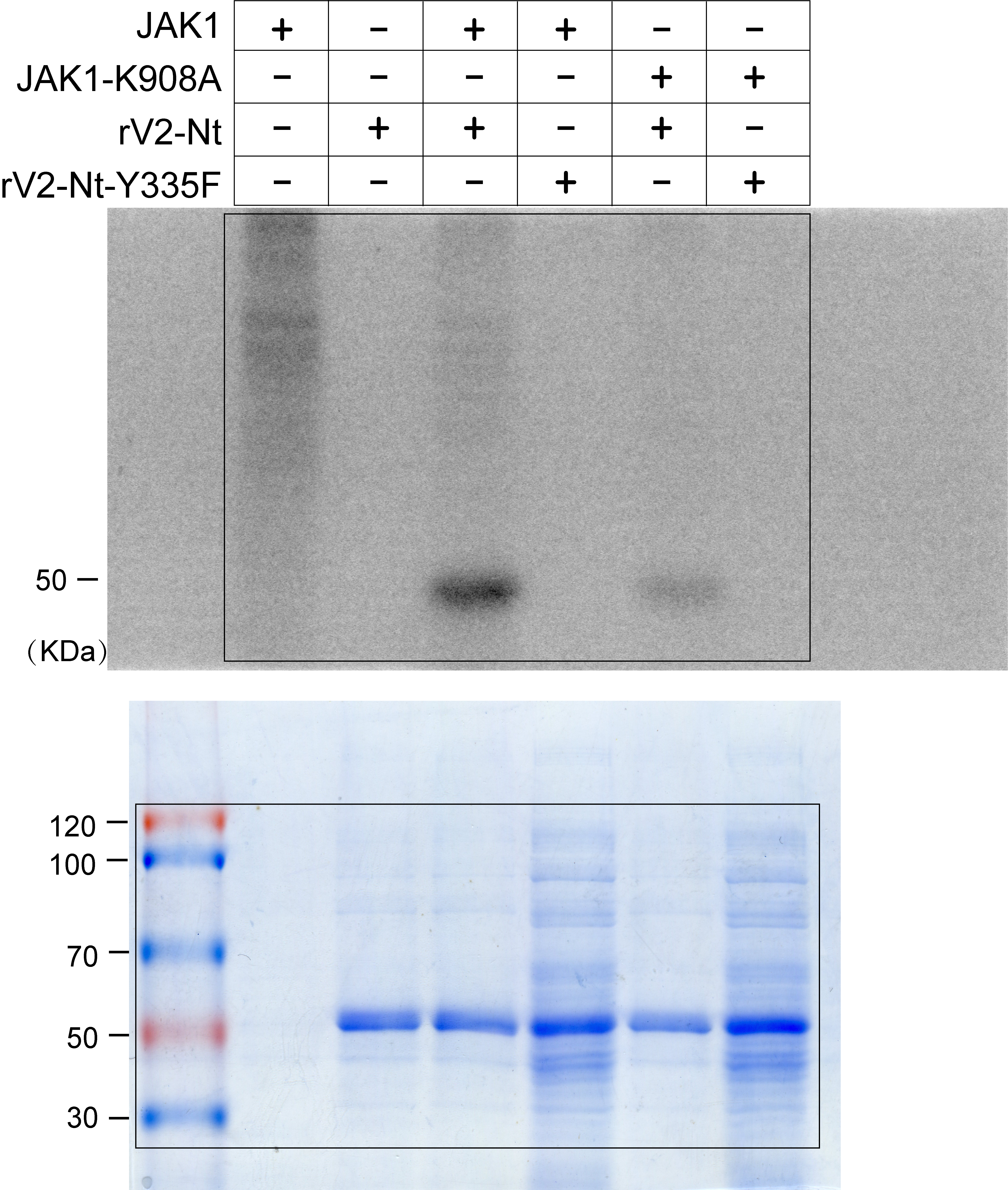

Supplement: Figure 4—source data 1. [file elife-78301-fig4-data1.zip › Figure 4 ¿C data source 1.tif]

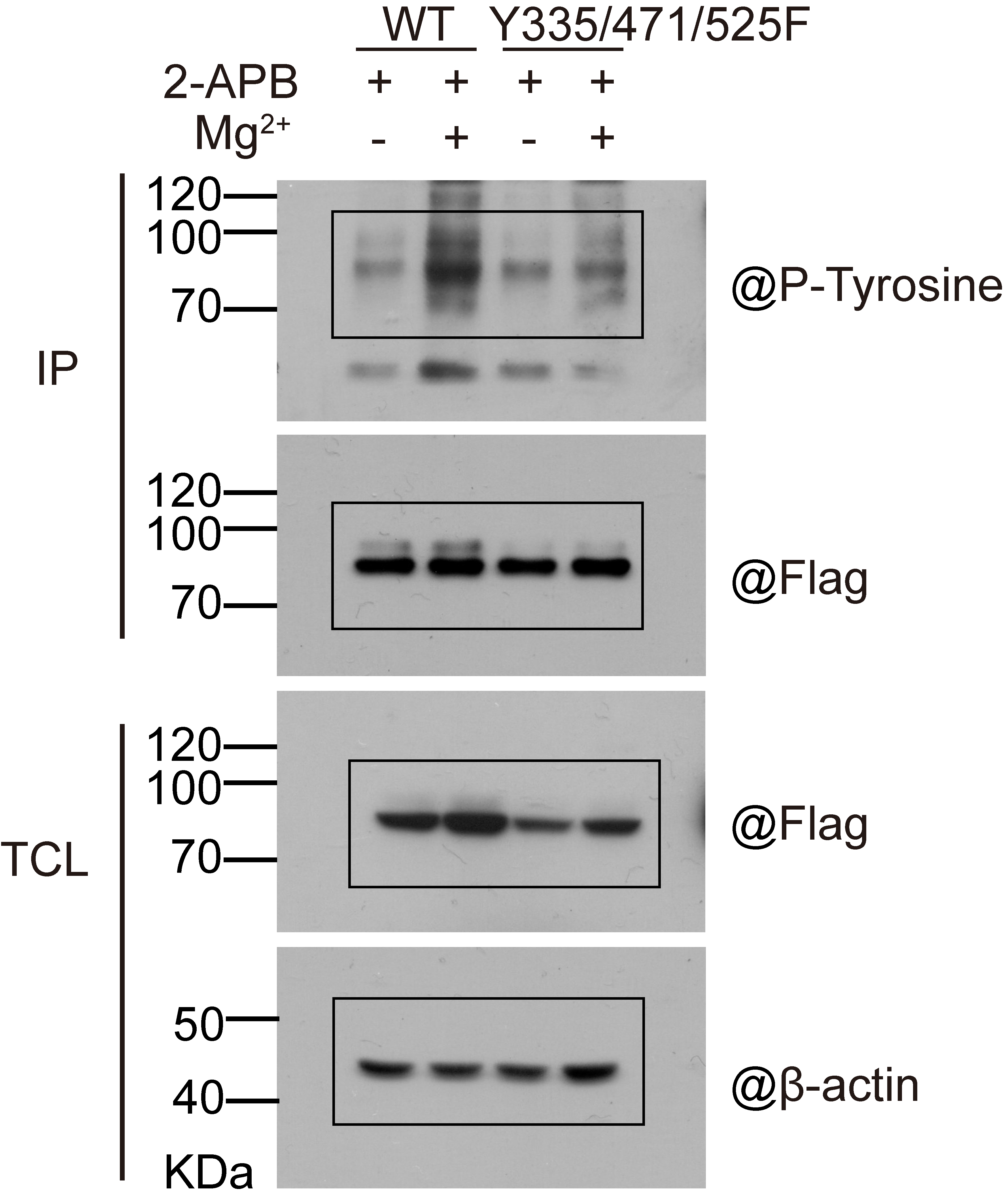

Supplement: Figure 4—source data 2. [file elife-78301-fig4-data2.zip › Figure 4 ¿C data source 2.tif]

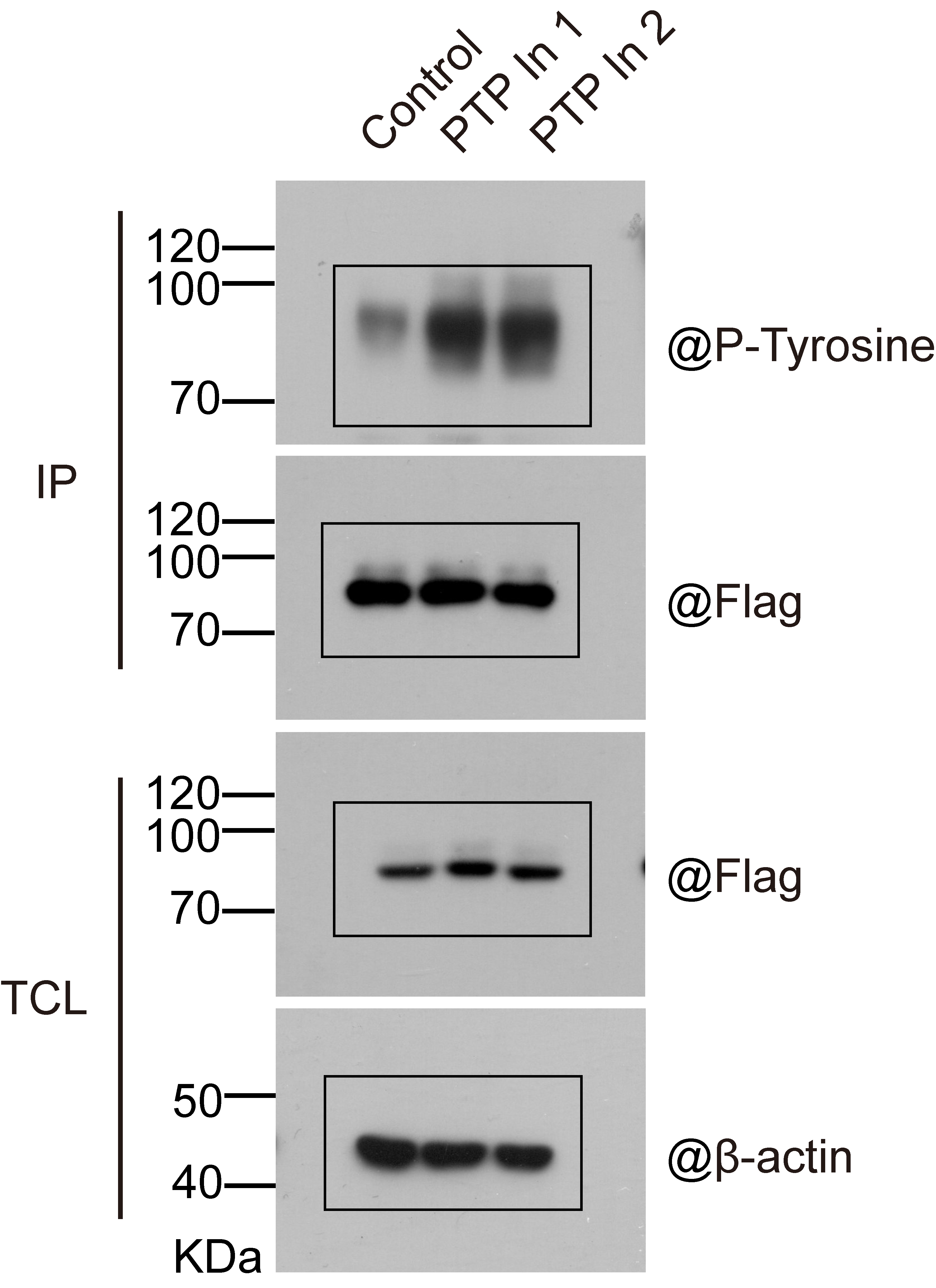

Supplement: Figure 5—source data 1. [file elife-78301-fig5-data1.zip › Figure 5 ¿C data source 1.tif]

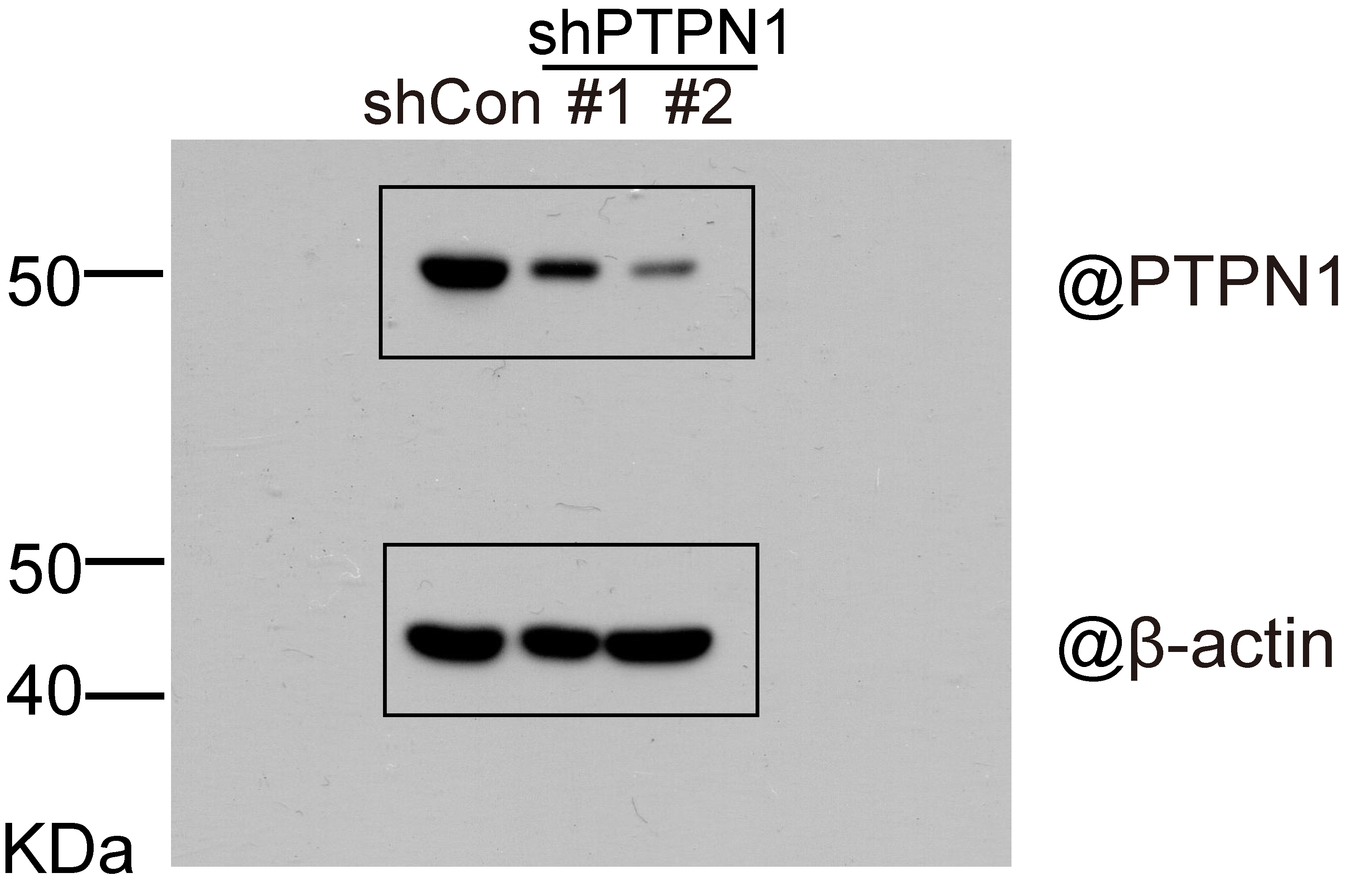

Supplement: Figure 6—source data 1. [file elife-78301-fig6-data1.zip › Figure 6 ¿C data source 1.tif]

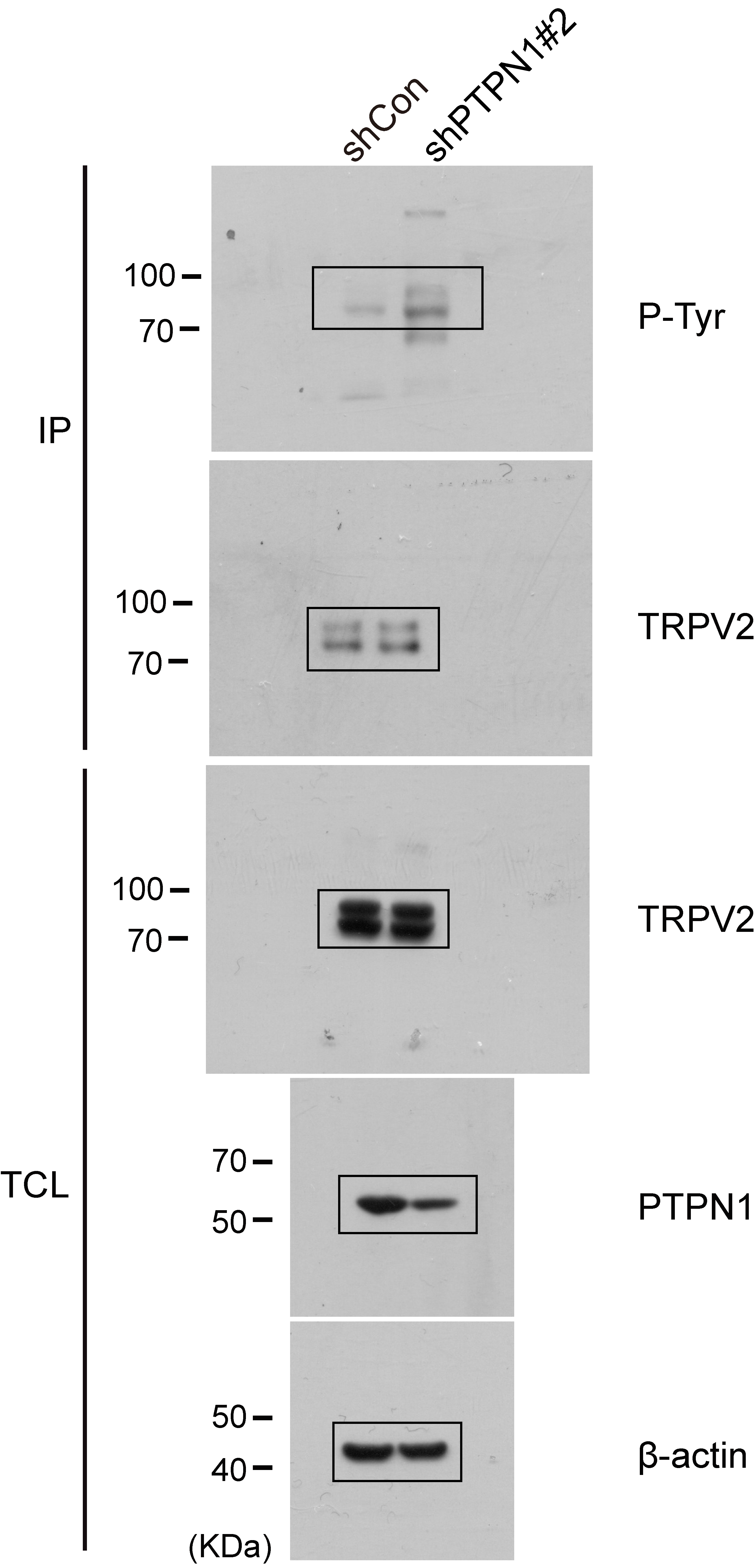

Supplement: Figure 6—source data 2. [file elife-78301-fig6-data2.zip › Figure 6 ¿C data source 2.tif]
